# Partial Atrioventricular Septal Defect Repair through Right Thoracotomy in A 73-Year-Old Woman

**DOI:** 10.70352/scrj.cr.25-0393

**Published:** 2025-10-15

**Authors:** Yuta Kitagata, Masaru Yoshikai, Taro Nakatsu, Etsuro Suenaga

**Affiliations:** 1 Department of Cardiovascular Surgery, Kansai Electric Power Hospital, Osaka, Osaka, Japan; 2 Department of Cardiovascular Surgery, Shin Koga Hospital, Kurume, Fukuoka, Japan

**Keywords:** minimally invasive cardiac surgery, partial atrioventricular septal defect, fresh autologous pericardium

## Abstract

**INTRODUCTION:**

While surgical treatment for partial atrioventricular septal defect (AVSD) is commonly performed in pediatric patients, its application is relatively rare in older patients.

**CASE PRESENTATION:**

A 73-year-old woman was referred to our hospital for evaluation of shunt blood flow caused by a partial atrioventricular septal defect and left atrioventricular valve regurgitation that caused right heart overload and cardiomegaly. She underwent minimally invasive atrial septal defect closure and left atrioventricular valve repair through right-sided thoracotomy.

**CONCLUSIONS:**

We report a successful case of minimally invasive surgery in an older patient with a partial atrioventricular septal defect.

## Abbreviations

ADLs
: activities of daily living
AVSD
: atrioventricular septal defect
LAVV
: left atrioventricular valve
PFO
: patent fossa ovalis
Qp/Qs
: pulmonary blood flow/systemic blood flow ratio

## INTRODUCTION

While surgical treatment for partial AVSD is commonly performed in pediatric patients,^1)^ its application is relatively rare in older patients.^2,3)^ Herein, we present a case of minimally invasive surgical treatment for partial AVSD in an older woman.

## CASE PRESENTATION

**Fig. 1:**
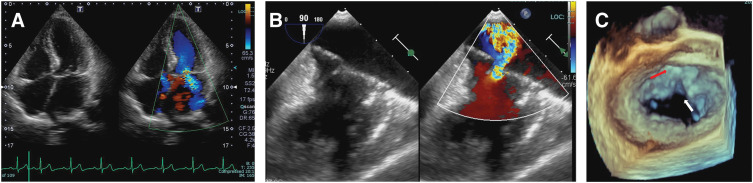
Preoperative echocardiography showing (**A**) the ostium primum defect and the absence of the ventricular septal defect, (**B**) left atrioventricular valve regurgitation. (**C**) 3D image showing the cleft of the left atrioventricular valve (white arrow) and atrial septal defect (red arrow).

A 73-year-old woman with no significant medical history was referred to our hospital with abnormal heart sounds characterized by a systolic murmur (Levine grade 2/6) and cardiomegaly (cardiothoracic ratio 0.63) observed on chest radiography. Transesophageal echocardiography revealed a partial AVSD (**Fig. 1**) with a PFO. It led to an increased Qp/Qs of 1.6 and right heart overload, with a transtricuspid valve pressure gradient of 34 mmHg. Moderate LAVV regurgitation is caused by a cleft between the left superior and inferior bridging leaflets. Arrhythmia and conduction disturbances were not observed on electrocardiography. She preferred right thoracotomy approach with a small incision over a conventional full sternotomy for cosmetic reasons.

**Fig. 2:**
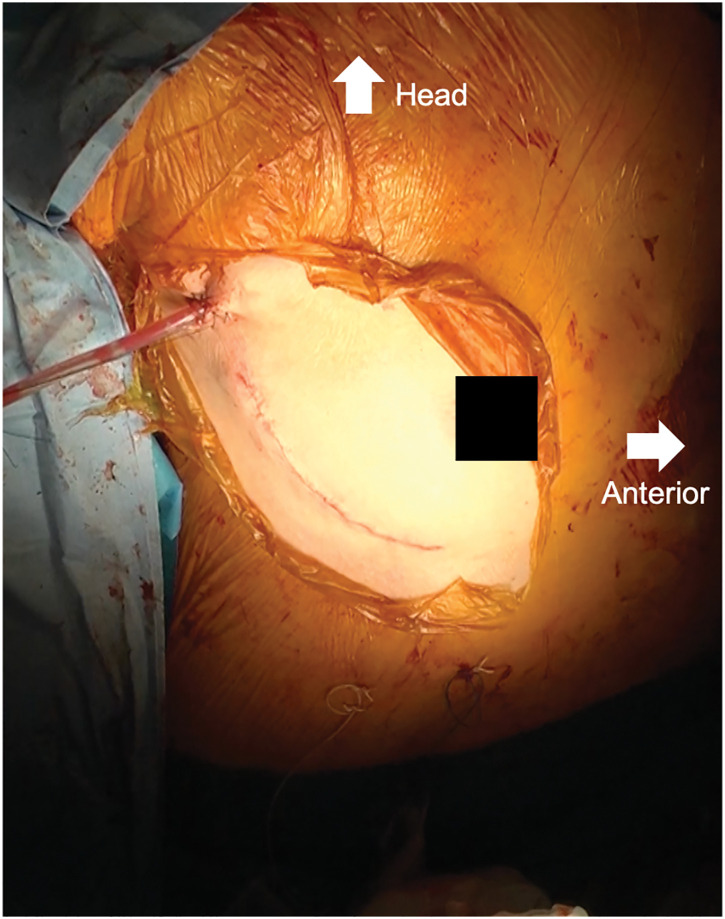
Intraoperative photograph showing the skin incision used for MICS. A right posterolateral thoracotomy was performed through the 4th intercostal space. The incision is located along the submammary line, providing excellent cosmetic outcomes while allowing adequate exposure for intracardiac procedures. MICS, minimally invasive cardiac surgery

**Fig. 3:**
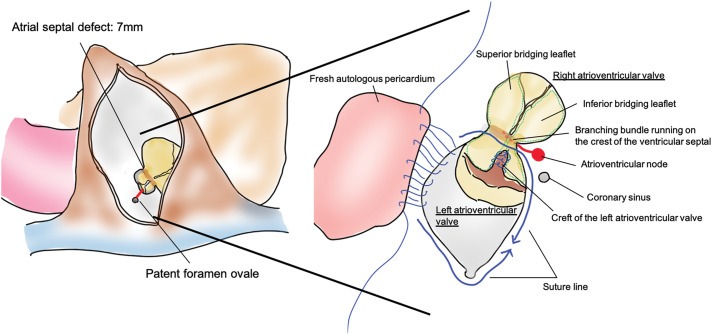
An incision (red line) was made in the atrial septum between the patent fossa ovalis to secure a surgical field to repair the valve, and the cleft was closed by a single 5-0 polypropylene suture, while the atrial septal defect was closed using fresh autologous pericardial tissue.

A 10-cm right thoracic anterolateral skin incision was made, and the chest was opened through the 4th intercostal space (**Fig. 2**, **Supplementary Video**). Cardiopulmonary bypass was performed via right femoral arterial perfusion and 2-sided drainage (the inferior caval vein through the right femoral and jugular veins), and aortic cross-clamping was achieved through the same incision used for the right intercostal thoracotomy. The ostium primum defect was observed through a right atrial incision, and the defect measured 7 mm in diameter. The fossa ovalis was normally formed and patent. To repair the LAVV, an incision was made in the atrial septum between the PFO and ostium primum defect to create a clear view to observe the LAVV. The cleft in the LAVV between the left superior and inferior bridging leaflets was closed using single 5-0 polypropylene sutures to eliminate regurgitation. Finally, the ostium primum defect extending to the PFO was closed using fresh autologous pericardium measuring 2 × 3 cm, keeping the coronary sinus positioned on the right atrial side with careful attention to avoid damaging the conduction system. A continuous suture is placed at the base of the LAVV, particularly at the inferior bridging leaflet. A saline test was subsequently performed to confirm that the regurgitation was adequately controlled. The suture line was placed on the left side of the ventricular septal crest to avoid bundle branches responsible for conduction (**Fig. 3**). The total operative time was 3 hours 37 minutes, with a cardiopulmonary bypass time of 122 minutes and an aortic clamp time of 94 minutes. The patient recovered smoothly and was discharged 8 days postoperatively. Follow-up echocardiography revealed mild LAVV regurgitation, no blood flow through the atrial septum, and no conduction disturbances. The sizes of the left and right atria were reduced, and the transtricuspid valve pressure gradient decreased from 34 to 22 mmHg.

## DISCUSSION

Minimally invasive cardiac surgery has been successfully performed in pediatric AVSD patients^4)^ and is considered feasible in older patients.^5)^ The objective of treating partial AVSD is to prevent heart failure arising from shunt flow and the risk of embolization due to right–left shunts. However, in older patients, conventional cardiac surgery with a median sternotomy introduces concerns regarding diminished ADLs because it is associated with various complications. Therefore, employing minimally invasive cardiac surgery, particularly in older populations, is highly advantageous because it permits treatment while preserving ADLs and is appealing from a cosmetic perspective. In the present case, the patient underwent surgery via a small incision in the right intercostal space, which facilitated a short hospital stay and discharge without compromising functional independence. Furthermore, the incision remained inconspicuous even under a wide-necked attire, contributing to the patient's overall satisfaction with the chosen approach.

Several important factors must be considered when performing this procedure. First, it is advisable to position the incision line slightly more anteriorly to facilitate access through the right atrial incision because LAVV intervention in partial AVSD is typically performed through a transseptal approach via the right atrium. Second, in cases in which the ostium primum defect was too small to permit a clear view, extending the ostium primum defect to the PFO enabled adequate visualization of the LAVV. Finally, although the incision from the ostium primum defect to the PFO in this case did not pose a risk of injury, caution was exercised during the ostium primum defect closure using autologous pericardial tissue to avoid damage to the conduction system. In cases of partial AVSD, the atrioventricular node is commonly displaced more posteriorly than usual and the subsequent long bundle branch runs through the crest of the ventricular septum. To ensure that the surgical protocol adheres to these points, accurate preoperative anatomical evaluation is necessary.

Annuloplasty with an artificial ring or band to mitigate the recurrence of LAVV regurgitation should be considered in individual cases, as demonstrated by Zhou et al.^6)^ who reported a lower recurrence rate with annuloplasty for LAVV regurgitation associated with partial AVSD. In this case, the valve ring was deemed sufficiently small to render annuloplasty unnecessary. However, annuloplasty warrants consideration in cases where valve ring enlargement occurs due to left atrial enlargement.

## CONCLUSIONS

Considering these points, this minimally invasive approach has proven to be effective in treating partial AVSD in older patients.

## SUPPLEMENTARY MATERIALS

Supplementary Video Partial atrioventricular defect with left atrioventricular valve regurgitation repaired using a minimally invasive method.

## DECLARATIONS

### Availability of data and materials

All relevant data have been provided within the manuscript.
